# The Influence of Gender and Anthropometry on Haemodynamic Status at Rest and in Response to Graded Incremental Head-Up Tilt in Young, Healthy Adults

**DOI:** 10.3389/fphys.2016.00656

**Published:** 2017-01-04

**Authors:** Delphine Sarafian, Jennifer L. Miles-Chan

**Affiliations:** Laboratory of Integrative Cardiovascular and Metabolic Physiology, Division of Physiology, Department of Medicine, University of FribourgFribourg, Switzerland

**Keywords:** head-up tilt, gender, haemodynamics, blood pressure regulation, sex difference

## Abstract

The body's ability to rapidly and appropriately regulate blood pressure in response to changing physiological demand is a key feature of a healthy cardiovascular system. Passively tilting the body, thereby changing central blood volume, is a well-recognized and controlled method of evaluating this ability. However, such studies usually involve single tilt angles, or intermittent tilting separated by supine, resting periods; valuable information concerning the adaptive capacity of the regulatory systems involved is therefore currently lacking. Furthermore, despite increasing recognition that men and women differ in the magnitude of their haemodynamic response to such stimuli, little is known about the degree to which gender differences in body composition and anthropometry influence these regulatory pathways, or indeed if these differences are apparent in response to graded, incremental tilting. In the present study we measured, in 23 young, healthy adults (13 men, 10 women), the continuous beat-to-beat haemodynamic response to graded, incremental tilting (0°, 20°, 40°, 60°, and back to 40°) with each tilt angle lasting 16 min. On average, we observed increases in heart rate (+41%), blood pressure (+10%), and total peripheral resistance (+16%) in response to tilting. However, whilst men showed an immediate decrease in cardiac output upon tilting (−8.9%) cardiac output in women did not change significantly from supine values. Interestingly, the decrease in stroke volume observed in women was significantly less than that observed in men (−22 vs. −36%, *p* < 0.05); although the present study could not determine if this difference was due to gender *per se* or due to differences in body size (in particular height) between the two gender groups. Such disparities in the magnitude of autonomic response may indicate (in the case of our gradual incremental tilt procedure) a better buffering capacity to progressive changes in central blood volume in women; which warrants further investigation, particularly in light of the well-recognized differences in cardiovascular disease risk between men and women.

## Introduction

The appropriate and effective regulation of blood pressure, in order to maintain homeostasis despite changing physiological requirements, is a fundamental aspect of cardiovascular health. It has long been recognized that young women have, in general, lower resting blood pressure than young men, concomitant with a higher prevalence of orthostatic intolerance (Robertson, 1999; Waters et al., 2002; Fu et al., 2004). However, it has only recently been recognized that men and women regulate blood pressure through different physiological mechanisms—with young women generally showing enhanced parasympathetic (vagal) input to cardiac regulation, compared to the predominance of sympathetic vascular regulation observed in men (Evans et al., 2001; Hart et al., 2009, 2012; Kim et al., 2011; Joyner et al., 2015; Reulecke et al., 2016).

Passive tilting of the body (typically head-up), is widely used to investigate cardiovascular reflexes and regulatory capacity by altering blood distribution from the center to the periphery. However, whilst a number of studies have now explored gender differences using a single tilt angle (typically between 60° and 80°) compared to supine (Fu et al., 2005; Ndayisaba et al., 2015; Kangas et al., 2016; Patel et al., 2016; Reulecke et al., 2016), fewer studies have investigated these differences in response to graded, incremental tilting (i.e., no return to supine between each tilt angle (Shoemaker et al., 2001). Such studies are warranted as they may provide important information regarding response thresholds and possible gender differences in such thresholds.

Furthermore, it is important to note that little information is available concerning the role that anthropometric differences may play in the relationship between gender and blood pressure regulation. Indeed, we know that women typically have a smaller body size than men, with a higher proportion of fat to fat-free mass, which have been suggested to influence stroke volume and peripheral blood flow, respectively (Joyner et al., 2015). However, whether or not the variability in blood pressure regulation observed between men and women lies more in differences in body size/composition than in gender *per se* has not been fully explored.

With these limitations in our current understanding of the relationship between gender and blood pressure regulation, we aimed to assess the haemodynamic response to graded, incremental head-up tilting in a group of healthy, young adults, and to determine the degree to which any apparent gender differences may be related to differences in anthropometry and body composition.

## Materials and methods

### Subjects

23 healthy, young adults (10 women, 13 men; age 25.0 ± 0.5 y) participated in the present study. This sample size was derived using an online power calculator (http://www.statisticalsolutions.net/pssTtest_calc.php) and based on the following: (i) resting values from previous studies of our local young adult population; (ii) haemodynamic response observed by others using a similar study protocol (Shoemaker et al., 2001); and (iii) an error probably (α) of 0.05 and power (1-β) of 0.80. These calculations indicated a necessary sample size of 8–10 subjects in each gender group. All subjects were Caucasian, non-obese (BMI < 26 kg/m^2^) and weight-stable, with less than 3% body weight variation in the 6 months preceding the study. Smokers, pregnant, or breast-feeding women, individuals taking medication, and those with any blood pressure abnormalities or cardiometabolic disease were excluded. Women were only tested during the follicular phase of their menstrual cycle. Prior to the day of testing, participants visited the laboratory in order to complete a questionnaire regarding their lifestyle (including physical activity level, caffeine intake and sleep quality) and medical history, and to familiarize themselves with the experimental procedure and equipment. All participants were requested to avoid physical activity, caffeine, and dietary supplements in the 24 h prior to testing. This study was carried out with the approval of the Fribourg state ethical review board, and all subjects gave written informed consent in accordance with the Declaration of Helsinki.

### Anthropometric and body composition measurements

Before anthropometric measurement, subjects were requested to empty their bladder. Body weight, height, and sitting height were measured to the nearest 0.1 kg and 1 mm respectively, using a mechanical column scale with integrated stadiometer (Seca model 709, Hamburg, Germany). Body composition was then assessed using a multi-frequency bioelectrical impedance analysis (Inbody 720, Biospace Co., Ltd, Seoul, Korea), and waist circumference and abdominal fat percentage by bioelectrical impedance analysis using ViScan (Tanita Corporation, Tokya, Japan); which has been shown to be accurate both for the measurement of waist circumference (Schutz et al., 2012) and for predicting total abdominal fat when validated against Magnetic Resonance Imaging techniques (Browning et al., 2010; Thomas et al., 2010). Body surface area was calculated according to Mosteller's formula (Mosteller, 1987).

### Experimental design

All experiments were conducted in the morning (08:30) after a 12 h overnight fast, in a quiet room at a comfortable ambient temperature (21–23°C). Monitoring electrodes were placed on the subject's neck and trunk (as described below) and the subject was then asked to lie supine on a clinical tilt table (Model 2900-00; CNSystems, Medizintechnik, Graz, Austria) with integrated foot support. Subjects were positioned such that their feet were in contact with the foot support, with straps placed to secure the subject at the level of the hips. In order to minimize skeletal muscle work and movement artifacts, subjects were encouraged to avoid movement and remain relaxed throughout the test. During the whole experiment, the participants were able to watch calm movies or documentaries to prevent sleeping and avoid boredom.

After a baseline measurement period of 40–45 min in the supine position, the subjects were passively tilted in increasing increments of 20 degrees (i.e., supine, 20°, 40°, 60°), remaining at each head-up tilt (HUT) angle for 16 min. The motorized tilt table achieved each 20° of tilt within 4–5 s. The subjects were then returned to an angle of 40° for a further 16 min, allowing us to determine the reversibility of the haemodynamic changes observed. Incremental graded tilting was utilized so as to compare the steady state changes in cardiovascular parameters consecutive to a slow gradual tilt and thereby the temporal adaptation of the body to progressive fluid shift (from thorax to lower limbs). Furthermore, by avoiding large changes in tilt angle (such as could occur if each tilt were separated by a supine period, or a single large tilt angle were used) we aimed to avoid issues of over-stimulation, whereby subtle changes/adaptations may be masked. It should also be noted that: (i) a return to 40° at the end of the experimental protocol (rather than supine or 20°) was chosen after a pilot study found that returning directly from 60° to supine was not well tolerated by the subjects (discomfort and altered sensation of body position in the horizontal plan); and (ii) we chose to monitor the variables for 16 min at each tilt angle to ensure we were monitoring a steady-state response and to minimize movement artifacts.

### Continuous haemodynamic monitoring

Beat-to-beat cardiovascular recordings were performed using a Task Force Monitor (TFM) (CNSystems), with data sampled at a rate of 1000 Hz, as previously described (Girona et al., 2014; Miles-Chan et al., 2015). Briefly, ECG/Impedance electrodes were positioned together with upper arm and finger BP cuffs. Electrode strips were placed at the neck and thoracic regions, the latter specifically midclavicular at the xiphoid process level (Standard electrode kit, CNSystems). BP was monitored continuously using the Penaz principle from either the index or the middle finger of the right hand and was calibrated to oscillometric brachial BP measurements on the contralateral arm. During the experiment, the right hand and finger cuff were maintained at heart level, on an adjustable side board attached to the tilt table, in order to avoid hydrostatic pressure artifacts.

Impedance cardiography measurements, in which the changes in thoracic impedance are converted to reflect changes in thoracic fluid content over time, were performed based on the original Kubicek approach (Kubicek et al., 1966, 1970) but using an improved estimate of thoracic volume (Fortin et al., 2006), which allows calculation of cardiac stroke volume (SV). Heart rate (HR) was calculated from the appropriate RR-Interval (RRI). Cardiac output (CO) was computed as the product of SV and HR. Mean arterial BP (MAP) was calculated from diastolic BP (DBP) and systolic BP (SBP) as follows: MAP = DBP + ⅓(SBP − DBP). Total peripheral resistance (TPR) was calculated as MAP/CO. Indices of SV (SI), CO (CI), and TPR (TPRI) were calculated by dividing each of these variables by body surface area.

### Autonomic assessment

Sympathovagal balance was evaluated by power spectrum analysis of HR and BP variability (Hilz and Dütsch, 2006). High frequency (HF: 0.15–0.40 Hz) power components of RRI (HF_RRI) and low-frequency (LF: 0.04–0.15 Hz) power components of DBP (LF_DBP) and SBP (LF_SBP) were assessed. Baroreflex sensitivity (BRS) was assessed using the sequence method from spontaneous fluctuations in HR and BP, as previously described (Bertinieri et al., 1985).

### Data and statistical analysis

All data are presented as Mean ± SEM unless otherwise stated, and were analyzed using the computer software STATISTIX 8 (Analytical Software, St. Paul, Minnesota, USA). Differences in baseline haemodynamic and anthropometric characteristics between genders were assessed by one-way ANOVA, and lifestyle parameters assessed by chi-square test. The mean of the last 4 min at each tilt (and the corresponding delta from baseline) were analyzed by repeated-measures ANOVA followed by Dunnett's multiple comparison tests. Relationships between the response of these haemodynamic variables and anthropometric characteristics were initially assessed by Pearson's correlation. Subsequently, positively-correlated anthropometric characteristics were entered into a stepwise linear regression (forward selection) analysis to determine the best predictive model for each haemodynamic variable.

## Results

### Anthropometry, body composition, and lifestyle

The anthropometric and body composition characteristics of the study population are shown in Table 1. On average, the women were shorter and lighter than the men, which was reflected in a lower average body surface area. However, the ratio of sitting height to height (Cormic index) did not differ between the two genders, and the small difference in BMI was at the limit of statistical significance (*p* = 0.05). Women showed a greater total body fat mass (both in terms of absolute mass and percentage fat), and a higher percentage of body fat in the trunk. However, in contrast both visceral fat area and waist circumference were lower in women than men. In terms of lifestyle parameters (assessed by questionnaire), there were no differences between men and women in terms of physical fitness levels, caffeine intake, or sleep quality.

**Table 1:** **Anthropometric characteristics of the study population**.

		**Men**		**Women**		***p-value***
		**mean**	***SEM***	**mean**	***SEM***	
Age	[y]	25.4	0.8	24.4	0.7	NS
Weight	[kg]	74.5	1.8	56.0	1.8	<0.001
Height	[cm]	180.9	1.7	161.4	1.4	<0.001
BMI	[kg/m^2^]	22.8	0.3	21.5	0.6	0.05
Sitting Height	[cm]	91.7	0.7	83.3	0.9	<0.001
Cormic Index	[1]	0.507	0.004	0.516	0.004	NS
Body Surface Area	[m^2^]	1.94	0.03	1.58	0.03	<0.001
Waist Circumference	[cm]	85.3	1.6	76.2	1.9	<0.005
Fat Free Mass	[kg]	64.3	1.4	42.5	1.4	<0.001
Skeletal Muscle Mass	[kg]	36.7	0.8	23.3	0.8	<0.001
Body Fat Mass	[kg]	10.4	1.0	13.6	1.0	<0.05
Body Fat	[%]	13.8	1.1	24.0	1.4	<0.001
Trunk Lean Mass	[kg]	28.0	0.7	18.2	0.6	<0.001
Trunk Fat Mass	[kg]	5.04	0.65	6.40	0.56	NS
Trunk Fat	[%]	14.9	1.6	25.8	1.6	<0.001
Abdominal Fat	[%]	15.8	1.2	24.2	1.2	<0.001
Visceral Fat Area	[cm^2^]	55.7	3.9	43.2	3.8	<0.05

*BMI, body mass index; NS, not significant. Cormic index calculated as ratio of sitting height to height. P-value indicates significance of difference as determined by one-way ANOVA*.

### Resting cardiovascular parameters

**Figure 1:**
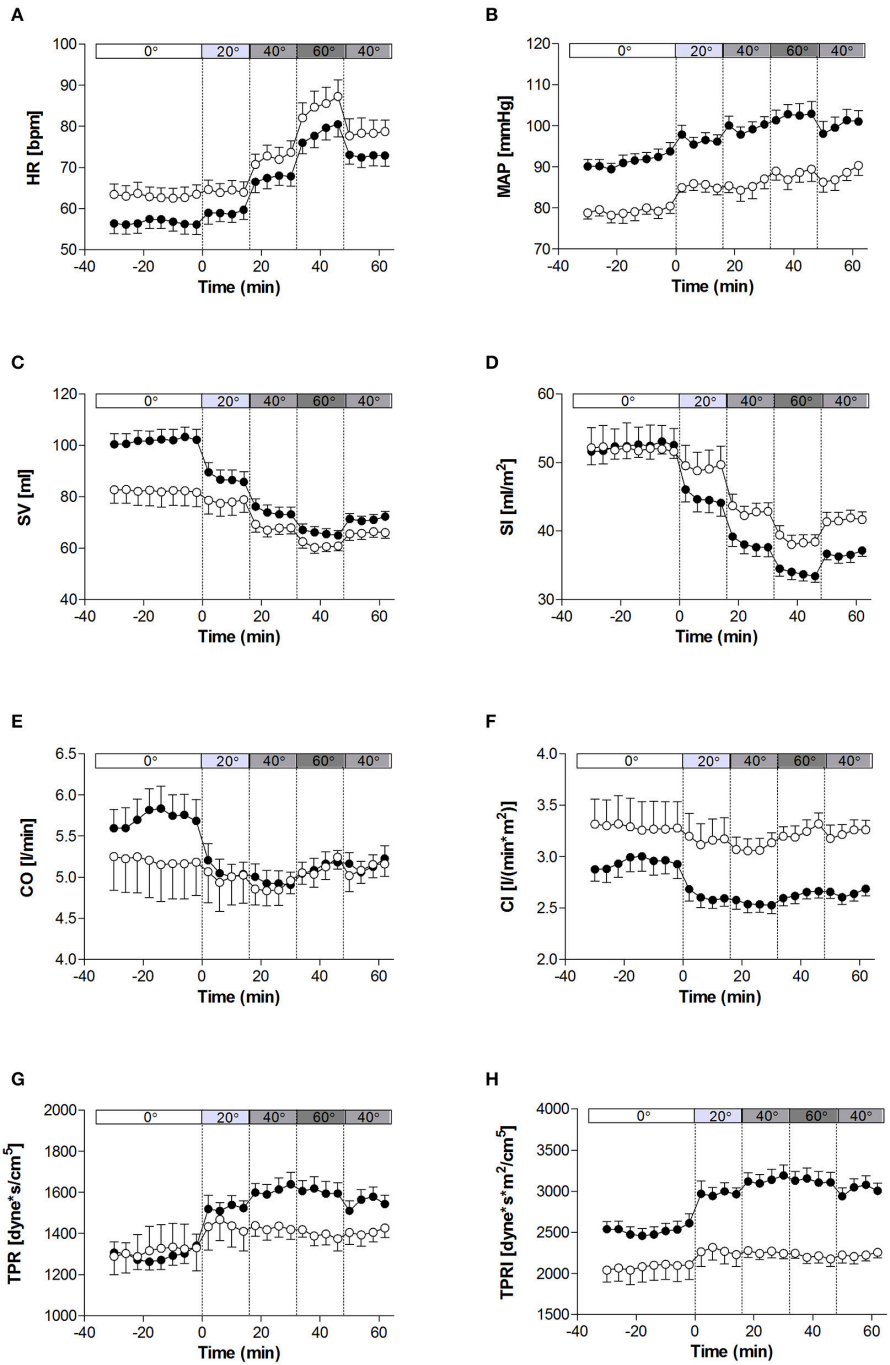
**Hemodynamic measures at rest and in response to graded, incremental tilt in healthy young adults. (A)** HR, heart rate; **(B)** MAP, mean arterial blood pressure; **(C)** SV, stroke volume; **(D)** SI, stroke index; **(E)** CO, cardiac output; **(F)** CI, cardiac index; **(G)** TPR, total peripheral resistance; **(H)** TPRI, total peripheral resistance index. Values are mean ± SEM of 4 min of measurement. Men (*n* = 13) are represented by closed circles; women (*n* = 10) are represented by open circles. Tilt angle is indicated in the bar above each panel (i.e., 0°, 20°, 40°, 60°, and back to 40°).

**Figure 2:**
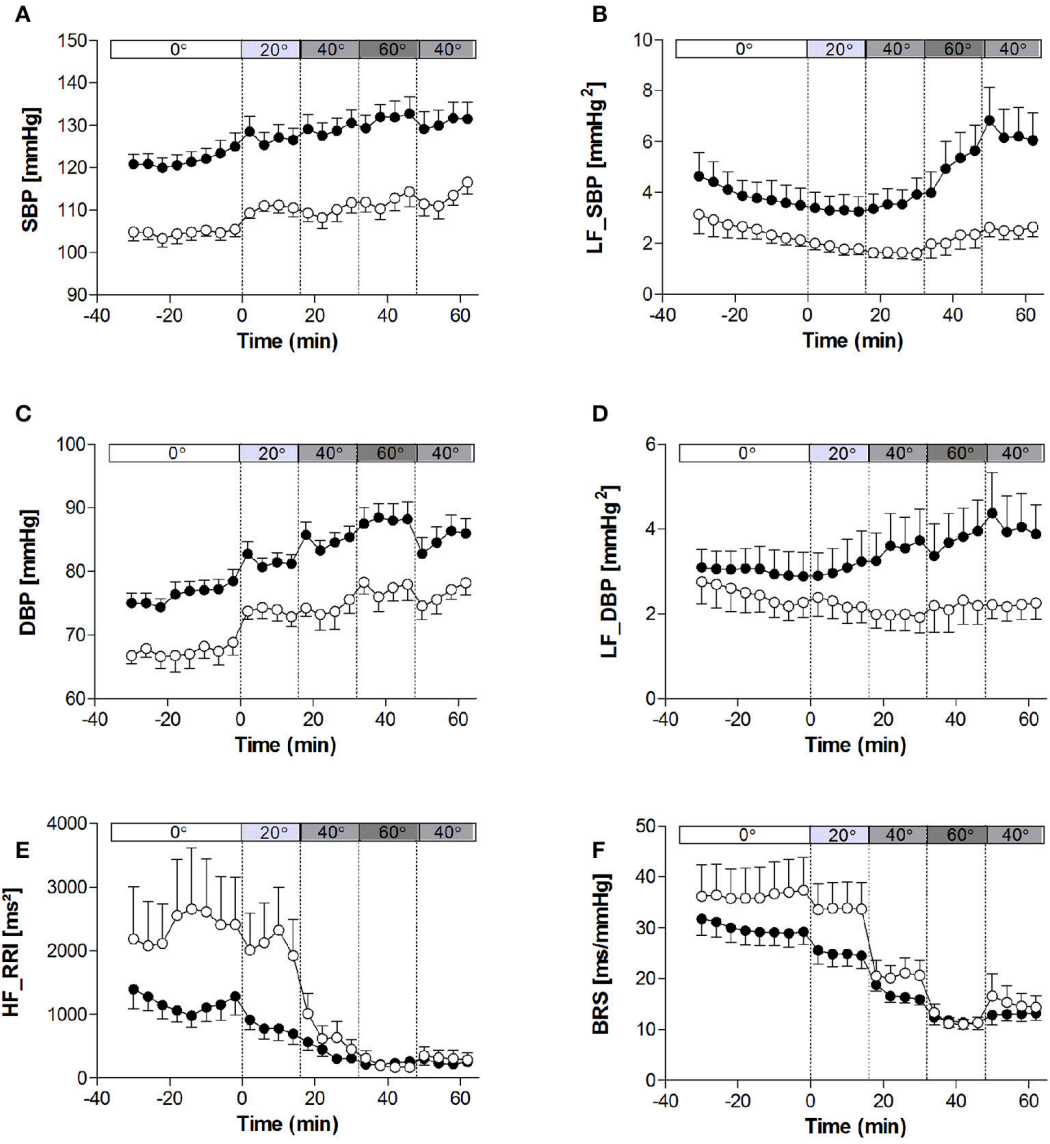
**Heart rate variability, blood pressure variability, and baroreflex sensitivity at rest and in response to graded, incremental tilt in healthy young adults. (A)** SBP, systolic blood pressure; **(B)** LF_SBP, low-frequency power component of SBP; **(C)** DBP, diastolic blood pressure; **(D)** LF_DBP, low-frequency power component of DBP; **(E)** HF_RRI, high frequency power component of R-R interval; **(F)** BRS, baroreflex sensitivity. Values are mean ± SEM of 4 min of measurement. Men (*n* = 13) are represented by closed circles; women (*n* = 10) are represented by open circles. Tilt angle is indicated in the bar above each panel (i.e., 0°, 20°, 40°, 60°, and back to 40°).

The haemodynamic characteristics of the study population at rest and in response to the graded tilt are summarized in Figures 1, 2. During the baseline supine period, heart rate was higher (Figure 1A; *p* < 0.05), and mean blood pressure lower (Figure 1B; *p* < 0.001) in women compared to men. This difference in blood pressure could be observed in both SBP (Figure 2A; *p* < 0.001) and DBP (Figure 2C; *p* < 0.005). Stroke volume was lower in women at rest (Figure 1C; *p* < 0.01), however there was no significant difference between the two genders at rest when adjusted for body surface area (i.e., stroke index, SI; Figure 1D). There were also no significant differences between men and women in terms of either CO (Figure 1E), CO adjusted for body surface area (cardiac index, CI; Figure 1F), TPR (Figure 1G), or BRS (Figure 2F). However, the mean TPR index (TPRI; Figure 1H) at rest was higher in men than women (*p* < 0.05).

### Cardiovascular response to tilting

**Figure 3:**
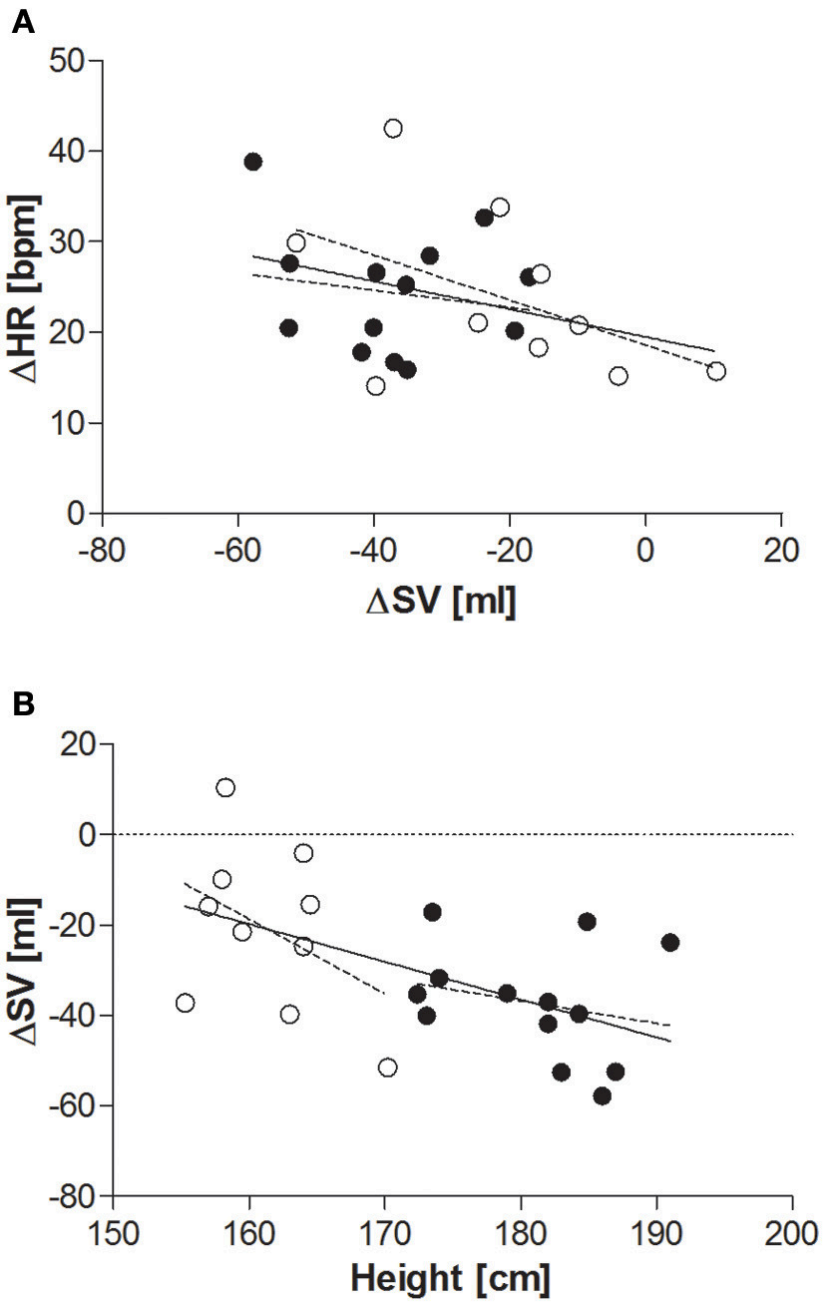
**(A)** Relationship between change in SV and change in HR (supine to 60° tilt). Men (*n* = 13) are represented by closed circles; women (*n* = 10) are represented by open circles. HR, heart rate (beats per minute); ΔHR, change in HR between supine and 60° head-up tilt; SV, stroke volume (ml); ΔSV, change in SV between supine and 60° head-up tilt. Pearson's correlation: all subjects: *r* = −0.339, *p* = 0.1; men: *r* = −0.177, *p* = 0.6; women: *r* = −0.489, *p* = 0.2. **(B)** Relationship between height and overall change in SV supine to 60° tilt. Men (*n* = 13) are represented by closed circles; women (*n* = 10) are represented by open circles. SV, stroke volume; ΔSV, change in SV between supine and 60° head-up tilt. Pearson's correlation: all subjects: *r* = −0.552, *p* < 0.01; men: *r* = −0.243, *p* = 0.4; women: *r* = −0.410, *p* = 0.2.

When comparing the last 4 min at each tilt angle, HR, MAP, SBP, and DBP all increased in response to the graded, incremental tilt (all *p* < 0.001), with no differential effect of gender. SV and SI showed significant effects due to both tilt angle (*p* < 0.001) and gender (*p* < 0.001), with significant decreases observed in men at 20° but in women, despite a downward trend seen at 20°, this difference only reached statistical significance after the 40° tilt. Overall, the magnitude of this decrease in SV (from supine to 60°) was greater in men than women (men: −37.2 ± 4.2 ml (−36%); women: −20.9 ± 4.8 ml (−22%); *p* < 0.05), but this difference did not persist when SV was adjusted for body surface area (SI). No significant correlation was observed between change in HR and change in SV across the tilting protocol (Figure 3A). Furthermore, CO and CI immediately and significantly decreased in men following the 20° tilt (*p* < 0.001), but in women did not differ significantly from supine values at any of the tilt angles. The same pattern was observed in terms of the high-frequency power component of RRI (HF_RRI; Figure 2E), and was mirrored by changes in TPR and TPRI; which both increased in men, but did not significantly differ from supine in women. The low-frequency components of SBP and DBP (Figures 2B,D, respectively) were both significantly lower in women than men throughout the tilting protocol. However, the only significant change noted in response to the tilting itself was a significant increase in LF_SBP in men during the 60° tilt relative to supine. Lastly, tilting caused a significant decrease in BRS (*p* < 0.001), with significant decreases observed in men at 20° but in women only from the 40° tilt onward.

Whilst MAP during the second 40° tilt period matched that of the first, CO and HR remained elevated and BRS remained depressed in both men and women during this second (post 60°) period.

### Correlations

Resting blood pressure was significantly positively correlated with weight, height, sitting height, body surface area, waist circumference, fat free mass, skeletal muscle mass, and trunk lean mass, and negatively correlated with percentage body fat (Table 2). However, following stepwise linear regression to remove auto-correlates, only height remained significantly predictive of MAP (Table 3).

**Table 2:** **Correlations (Pearson) between anthropometric characteristics and haemodynamic variables (at rest and in response to head-up tilt)**.

		**Resting (0°)**										**Change (0° vs. 60°)**	
		**MAP**		**SBP**		**DBP**		**HR**		**SV**		**SV**	
		***r***	***p***	***r***	***p***	***r***	***p***	***r***	***p***	***r***	***p***	***r***	***p***
Age	[y]	0.00	0.991	−0.07	0.766	0.06	0.795	−0.34	0.110	0.38	0.072	−0.26	0.225
Weight	[kg]	**0.71**	**<0.001**	**0.77**	**<0.001**	**0.60**	**0.002**	−**0.42**	**0.046**	**0.54**	**0.008**	−**0.40**	**0.061**
Height	[cm]	**0.74**	**<0.001**	**0.82**	**<0.001**	**0.60**	**0.002**	−**0.47**	**0.025**	**0.66**	**0.001**	−**0.55**	**0.006**
Body mass index	[kg/m^2^]	0.30	0.160	0.34	0.117	0.29	0.186	−0.21	0.346	0.10	0.660	0.08	0.713
Sitting height	[cm]	**0.68**	**<0.001**	**0.72**	**<0.001**	**0.58**	**0.004**	−0.39	0.071	**0.52**	**0.013**	−**0.45**	**0.035**
Cormic index	[1]	−0.39	0.076	−**0.52**	**0.012**	−0.22	0.324	0.26	0.249	−**0.45**	**0.035**	0.35	0.109
Body surface area	[m^2^]	**0.72**	**<0.001**	**0.79**	**<0.001**	**0.60**	**0.003**	−**0.44**	**0.034**	**0.58**	**0.004**	−**0.44**	**0.037**
Waist circumference	[cm]	**0.58**	**0.005**	**0.64**	**0.001**	**0.52**	**0.013**	0.02	0.931	0.37	0.088	−0.32	0.148
Fat free mass	[kg]	**0.71**	**<0.001**	**0.77**	**<0.001**	**0.57**	**0.005**	−**0.49**	**0.019**	**0.57**	**0.004**	−**0.46**	**0.028**
Skeletal muscle mass	[kg]	**0.69**	**<0.001**	**0.76**	**<0.001**	**0.56**	**0.005**	−**0.49**	**0.019**	**0.56**	**0.005**	−**0.45**	**0.032**
Body fat mass	[kg]	−0.16	0.467	−0.19	0.378	−0.06	0.781	0.28	0.204	−0.22	0.313	0.30	0.163
Body fat	[%]	−**0.48**	**0.021**	−**0.52**	**0.010**	−0.34	0.113	**0.45**	**0.031**	−**0.47**	**0.023**	**0.47**	**0.025**
Trunk lean mass	[kg]	**0.73**	**<0.001**	**0.81**	**<0.001**	**0.59**	**0.003**	−**0.43**	**0.040**	**0.54**	**0.008**	−**0.45**	**0.032**
Trunk fat mass	[kg]	−0.05	0.823	−0.09	0.682	0.04	0.857	0.21	0.335	−0.11	0.602	0.20	0.357
Trunk fat	[%]	−0.40	0.056	−**0.46**	**0.026**	−0.27	0.213	0.40	0.058	−0.37	0.081	0.39	0.067
Visceral fat area	[cm^2^]	0.37	0.080	0.37	0.083	0.38	0.071	−0.12	0.593	0.29	0.184	−0.15	0.481

*Only haemodynamic variables with one or more statistically significant correlations with anthropometry are shown (i.e., no significant correlations were found in terms of cardiac output, total peripheral resistance, etc). These correlations are highlighted in bold*.

*BMI, body mass index; DBP, diastolic blood pressure; HR, heart rate; MAP, mean arterial pressure; SBP, systolic blood pressure; SV, stroke volume. Cormic index calculated as ratio of sitting height to height*.

**Table 3:** **Stepwise linear regression analysis (forward selection) of resting mean arterial pressure, heart rate, and stroke volume**.

**Step**	**Variable**	**Coefficient**	***T***	***P***	***R*****^2^**	**MSE**
**RESTING MEAN ARTERIAL PRESSURE**						
1	Constant	87.99	42.16		0.000	95.85
2	Constant	−24.60	−1.13		0.573	43.02
	Height	0.65	5.18	<0.0001		
**Excluded Variables**						
	Weight		0.47	0.647		
	Sitting Height		−0.19	0.851		
	Body Surface Area		0.18	0.856		
	Waist circumference		0.28	0.781		
	Fat Free Mass		−0.66	0.518		
	Skeletal Muscle Mass		−0.71	0.484		
	Body Fat [%]		0.85	0.405		
	Trunk Lean Mass		−0.04	0.971		
**RESTING HEART RATE**						
1	Constant	59.33	31.89		0.000	79.62
2	Constant	77.59	10.54		0.236	63.73
	Skeletal Muscle Mass	−0.59	−2.55	0.019		
**Excluded Variables**						
	Weight		0.65	0.524		
	Height		0.04	0.965		
	Body Surface Area		0.50	0.624		
	Fat Free Mass		−0.02	0.983		
	Body Fat [%]		0.62	0.543		
	Trunk Lean Mass		1.49	0.151		
**RESTING STROKE VOLUME**						
1	Constant	92.14	23.25		0.000	345.45
2	Constant	−88.66	−1.82		0.410	214.12
	Height	1.05	3.73	0.001		
3	Constant	−382.95	−2.92		0.545	173.56
	Height	3.45	3.32	0.004		
	Trunk Lean Mass	−5.03	−2.38	0.028		
**Excluded Variables**						
	Weight	−0.18	0.857			
	Sitting Height	−0.72	0.484			
	Cormic Index	−0.65	0.521			
	Body Surface Area	−0.49	0.627			
	Fat Free Mass	0.36	0.720			
	Skeletal Muscle Mass	0.31	0.763			
	Body Fat [%]	−0.94	0.360			

*MSE, mean square error*.

Resting heart rate was significantly, negatively correlated with weight, height, body surface area, fat free mass, skeletal muscle mass, and trunk lean mass (Table 2); with only skeletal muscle mass remaining significant following stepwise linear regression (Table 3). Conversely, resting (supine) SV was positively correlated with weight, height, sitting height, body surface area, fat free mass, skeletal muscle mass, and trunk lean mass, and negatively correlated with percentage body fat, and Cormic index (Table 2); with height and trunk lean mass remaining significantly predictive following stepwise linear regression (Table 3).

Whilst none of the measured anthropometric or body composition variables were found to be predictive of change in HR or blood pressure during the graded incremental tilt, significant correlations were observed between a number of these variables and change in steady-state SV (Table 2)—with height the primary predictive variable, and body surface area also contributing to the resulting stepwise model (Table 4). However, the relationship between change in steady-state SV and height did not reach statistical significance when analyzing data within (rather than across) the two gender groups (Figure 3B).

**Table 4:** **Stepwise linear regression analysis (forward selection) of overall change in stroke volume (0°−60°)**.

**Step**	**Variable**	**Coefficient**	***T***	***P***	***R*****^2^**	**MSE**
1	Constant	−29.56	−8.05		0.000	296.70
2	Constant	111.63	2.26		0.291	220.92
	Height	−0.82	−2.86	0.010		
3	Constant	259.58	3.15		0.430	187.04
	Height	−2.87	−2.90	0.009		
	Body Surface Area	114.84	2.15	0.045		
**Excluded Variables**		***T***	***P***			
	Weight	−0.88	0.389			
	Sitting Height	0.69	0.498			
	Fat Free Mass	0.76	0.456			
	Skeletal Muscle Mass	0.78	0.444			
	Body Fat [%]	−0.55	0.590			
	Trunk Lean Mass	1.11	0.282			

*MSE, mean square error*.

No significant correlations were found between either CO or TPR and any of the measured anthropometric or body composition variables at rest, nor in response to the graded tilt.

## Discussion

The results of the present study offer a clear demonstration of the ability of healthy young individuals to respond appropriately to changing blood distribution so as to maintain blood pressure and blood supply. This can be seen by the comparatively large decreases observed at 60° tilt in SV (on average −36% and −22% for men and women, respectively) as compared to the change in MAP (+10%), with the decrease in central blood volume being largely buffered by a large increase in heart rate (+41%; +24 beats/min), and augmented TPR (+16%; +21% in men, +10% in women) such that CO remained fairly constant. However, despite HR and SV being closely, inversely related (Cote et al., 2012) no significant correlation between change in each of these variables over the tilting protocol was found—which is most likely due to the relatively small sample size of the present study (as discussed below), but could also be related to an apparent heterogeneity in the relationship between these two variables (Goswami et al., 2009), or intrinsic variability in haemodynamic adjustments occurring within the heart and lungs (Guazzi et al., 1995). Indeed, the change in CO observed during the 60° tilt (average: −1.7 ± 3.9%) was less than that observed by others (10–23%) (Tuckman and Shillingford, 1966; Hainsworth and Al-Shamma, 1988; Fu et al., 2005; Bundgaard-Nielsen et al., 2009). This apparent discrepancy is most likely due to the incremental nature of the tilting protocol used in the present study as opposed to the discontinuous protocols (i.e., each tilt separated by a supine rest) used in the latter studies, and hence our findings may be more representative of the *adaptive capacity* of the haemodynamic regulatory mechanisms involved.

Under resting, supine conditions, the women in the present study showed a higher average heart rate, and lower average blood pressure, stroke volume, and total peripheral resistance than their male counterparts; although these differences were most likely due to differences in skeletal muscle mass and height between the two groups rather than an effect of gender *per se*. Similarly, differences in the magnitude of changes in steady-state CO and TPR between men and women could be largely accounted for by differences in body surface area (i.e., when considering CI and TPRI).

A number of differences in the time-course of the haemodynamic response also appear to exist between the genders which cannot be explained by anthropometry alone. In particular, the majority of measured variables changed immediately upon tilting in men (i.e., between supine and 20°), but not until 40° in women. These changes are in parallel to BRS and vagal tone (as represented by HF_RRI) which remained elevated in women during the 20° but dropped dramatically at 40°. Taken together, these results indicate an asymmetry in the time course of parasympathetic response to graded tilt between men and women. The relative lack of change in the LF components of SBP and DBP observed in women while supine and across all tilt angles may be due to sex hormones, in particular estrogen, which is known to be sympatho-inhibitory, and inversely related to muscle sympathetic nerve activity (Joyner et al., 2015). In contrast, men displayed elevated sympathetic activity (as assessed by the LF power components of SBP and DBP), in line with a previous study showing significantly higher values of LF and total power in men than women when supine or standing upright (Barantke et al., 2008). Similarly, this agrees with the recent study of Reulecke et al. (2016), who investigated the effect of gender on the time-course of autonomic regulation during an orthostatic challenge (70°), and found that men showed an instantaneous increase in sympathetic activity which persisted across the entire orthostatic phase, and at a much higher level than that observed in women. The results of the present study therefore appear to support the suggestion of Kim et al. (2011) that, in comparison to men, women may rely more on baroreflex control of heart rate than on control of sympathetic vasomotor tone.

It should be noted, however, that there were some key limitations of this study, and as such these potential gender differences should be interpreted with caution. *Firstly*, the sample size of the present study, whilst in line with others investigating the effect of gender on the haemodynamic response tilting (Shoemaker et al., 2001; Fu et al., 2005; Patel et al., 2016; Reulecke et al., 2016), is not sufficient to draw any conclusions regarding the relative contribution of gender *per se* above that of gender-related differences in body size and/or composition. Further investigation is therefore warranted to confirm these results in a larger number of participants and across a larger range of body morphology. *Secondly*, whilst impedance cardiography has shown good correlations with both Doppler (Cybulski et al., 1993) and invasive techniques (Fortin et al., 2006) for the determination of SV, this assessment does require estimation of the electrical participating thoracic volume (a function of the subject's gender, height, and weight), and is therefore prone to error. Additional error may arise from small changes in the shape of the thorax or position of the heart that occur with changes in body posture.

Interestingly, there was a strong relationship observed between change in SV in response to tilting and height; with height and BMI together accounting for >40% of variability in delta SV. However, the present study could not determine if the difference in SV response observed between men and women was reflective of differences in body size (in particular height) between the two gender groups or due to gender *per se*. It would therefore be of interest to determine whether this relationship can also be observed within genders and/or across a larger range of heights, particularly given the well-established epidemiological link between short stature and risk for coronary heart disease (Paajanen et al., 2010). Furthermore, the smaller decreases in SV observed in women compared to men during the incremental head-up tilt—even when adjusted for differences in body size (i.e., stroke index)—are of particular interest, as they appear to be at odds with reports of an increased prevalence of orthostatic intolerance in young women (Robertson, 1999; Waters et al., 2002; Fu et al., 2004). This finding may indicate a better buffering capacity in response to slow progressive changes in central blood volume in women (as occurred with incremental tilting), and warrants further investigation, particularly in light of the well-recognized differences in cardiovascular disease risk between men and women (Hart et al., 2012).

## Author contributions

Both authors (DS and JM-C) performed the experiment and analyzed the data; JM-C wrote the manuscript; both authors (DS and JM-C) read and approved the final manuscript.

### Conflict of interest statement

The authors declare that the research was conducted in the absence of any commercial or financial relationships that could be construed as a potential conflict of interest.
